# Supplementary material for: Seven Epidemic Waves of COVID-19 in a Hospital in Madrid: Analysis of Severity and Associated Factors
Source: Viruses. 2023 Aug 30;15(9):1839. doi: 10.3390/v15091839 (PMC10538062; doi:10.3390/v15091839)
Supplement: Supplementary file 1 [file viruses-15-01839-s001.zip › viruses-2519200-supplementary.pdf]

Supplementary Materials:

Table S1. Study variables*.

Variable	Description	Units (International System), If Applicable
Admission date	Date of first visit to the Emergency Department	
Age	Patient's age at the time of admission	years
Sex	Male or Female	
Place of birth	Caucasian, North African, Latin American	
Hypertension	Yes or not	
Diabetes	Yes or not	
Cardiopathy	Ischemic heart disease or heart failure, yes or not	
COPD	chronic obstructive pulmonary disease, yes or not	
Asthma	Yes or not	
Oncological disease	Any oncological disease	
HIV	human immunodeficiency virus infection, yes or not	
Dementia	Yes or not	
Charlson	Charlson comorbidity index: predicts 10-year survival in patients with multiple comorbidities.	
Type of vaccine	Type of vaccine received against SARS-CoV-2 infection: BNT162b2 mRNA (Pfizer/BioNTech), mRNA-1273 (Moderna), ChAdOx1 nCoV-19/AZD1222 (AstraZeneca/Oxford), Ad26.COV2.S (Janssen)	
Vaccination	Vaccinated: BNT162b2 mRNA: two-dose schedule 21 d apart. mRNA-1273: two-dose schedule 28 d apart. ChAdOx1 nCoV-19/AZD1222: two-dose schedule 56-84 d apart. Ad26.COV2.S: single dose. 14 d period after second dose is required to considered patient complete vaccinated. Not vaccinated: patient has not received any vaccine dose or received an incomplete schedule or after receiving the complete schedule started with COVID-19 symptoms before 14 d apart from de last dose	
Chest X-ray at admission	No pneumonia, Unilateral pneumonia, Bilateral pneumonia	
First oxygen saturation	First oxygen saturation in the emergency department at admission (absolute value and categorical: less than 94% Yes or not)	%
Worst oxygen saturation	Worst oxygen saturation throughout the admission (absolute value and categorical: less than 94% Yes or not)	%

O2 requirements:<br>None		
Low O2 flow	Nasal cannula or ventiMask	
High O2 flow	Reservoir mask or high-flow nasal cannula	
Mechanical ventilation	intubation	
ICU admission	Yes or not	
Death in hospital	Yes or not, from any cause at 3 months after admission	
Hospital stay	From hospital admission to discharge	days
ICU stay	From ICU admission to discharge	Days
CRP	Highest C-reactive protein blood value	mg/L
IL-6[2]	Highest Interleukin 6 value	pg/ml
DD[3]	Higesth D-dimer value	ng/ml
Ferritin[4]	Highest ferritin value	ng/ml
Remdesivir	Wether the patient received it or not	
Steroids	Wether the patient received it or not	
Tocilizumab	Wether the patient received it or not	
Baricitinib	Wether the patient received it or not	
pLMWH	Wether the patient received it or not	

* ICU: intensive care unit; VM: ventiMask: pLMWH: Low molecular weight heparin
